# Linking clinical quality indicators to research evidence - a case study in asthma management for children

**DOI:** 10.1186/s12913-017-2324-y

**Published:** 2017-07-21

**Authors:** Miew Keen Choong, Guy Tsafnat, Peter Hibbert, William B. Runciman, Enrico Coiera

**Affiliations:** 1 Centre for Health Informatics, Australian Institute of Health Innovation, Faculty of Medicine and Health Sciences, Macquarie University, Sydney, Australia; 2 Centre for Population Health Research, University of South Australia, Adelaide, South Australia; 3 Australian Patient Safety Foundation, Adelaide, South Australia

**Keywords:** Quality indicators, Randomised controlled trial, Asthma, Child, Outcome measures

## Abstract

**Background:**

Clinical quality indicators are used to monitor the performance of healthcare services and should wherever possible be based on research evidence. Little is known however about the extent to which indicators in common use are based on research. The objective of this study is to measure the extent to which clinical quality indicators used in asthma management in children with outcome measurements can be linked to results in randomised controlled clinical trial (RCT) reports. This work is part of a broader research program to trial methods that improve the efficiency and accuracy of indicator development.

**Methods:**

National-level indicators for asthma management in children were extracted from the National Quality Measures Clearinghouse database and the National Institute for Health and Care Excellence quality standards by two independent appraisers. Outcome measures were extracted from all published English language RCT reports for asthma management in children below the age of 12 published between 2005 and 2014. The two sets were then linked by manually mapping both to a common set of Unified Medical Language System (UMLS) concepts.

**Results:**

The analysis identified 39 indicators and 562 full text RCTs dealing with asthma management in children. About 95% (37/39) of the indicators could be linked to RCT outcome measures.

**Conclusions:**

It is possible to identify relevant RCT reports for the majority of indicators used to assess the quality of asthma management in childhood. The methods reported here could be automated to more generally support assessment of candidate indicators against the research evidence.

**Electronic supplementary material:**

The online version of this article (doi:10.1186/s12913-017-2324-y) contains supplementary material, which is available to authorized users.

## Background

The drive to improve the quality and safety of healthcare has resulted in a proliferation of clinical quality indicators. Indicators are regularly used to assess the quality of care, and to identify and prioritise areas for improvement [1, 2]. A good indicator should say “as much about a system as possible in as few points as possible” [3]. It should thus be important, relevant, valid, reliable, meaningful and understandable [3, 4]. In addition, indicators should be easy to collect [5], and the costs of doing so should not outweigh any benefits [6]. For these requirements to be met, indicators should be supported by a strong base of research evidence [2, 7].

The most common approach to indicator development is based on their extraction from clinical guidelines [8], with the guidelines themselves based upon randomised controlled trials (RCTs) [9, 10]. Thus, the development of indicators typically requires a lengthy manual process of searching for and analysis of the research evidence underpinning guidelines. A lack of uniformity in reporting the rationale for selecting indicators also means that it may be difficult to know whether an indicator is solidly based on research evidence. A lack of rigour in indicator selection can introduce biases that limit their usefulness in measuring the quality of care [8].

The development of methods that assists in connecting candidate indicators with the research evidence should help increase the efficiency of indicator development, and may also improve confidence in the quality of studies based on indicators. There are a number of steps in the process of assessing the evidence base behind any given indicator, starting with searching for and then linking indicators to RCTs, and ending with an assessment of the strength of the evidence for the use of the indicator in those RCTs. Clinical indicators are measures of clinical activity results, and outcome measures serve a similar role in RCT reports. In this study we report on a standardised and simple method to link indicators to outcomes reported in clinical trials, using publicly available clinical concept resources.

As a case study to support the development of methods to improve the efficiency and accuracy of indicator development, we examine the relationship between national level clinical quality indicators for asthma management in pre-adolescent children in the UK and the USA and recent RCT reports. Asthma is a chronic respiratory disease that affects about 300 million people globally, with an estimated 250,000 deaths annually [11]. It is the most common chronic disease among children. There is currently no cure, but it can generally be managed and controlled with appropriate care (care in-line with clinical guidelines) [12–14]. Many indicators have been developed for asthma [15–19], and most are based on clinical practice guidelines. For example, the National Institute for Health and Care Excellence (NICE) quality standards for asthma are based on the British Thoracic Society/Scottish Intercollegiate Guidelines Network (BTS/SIGN) clinical guideline [12]. Indicators for children may also apply to older populations but there may be changes in indicators as individuals move into adolescence and then adulthood. In adolescence, individuals typically assume responsibility for their asthma care and potentially begin experimenting with higher-risk behaviors [20]. As a result non-adherence, morbidity and mortality appear to be greater among adolescents.

## Methods

**Fig. 1:**
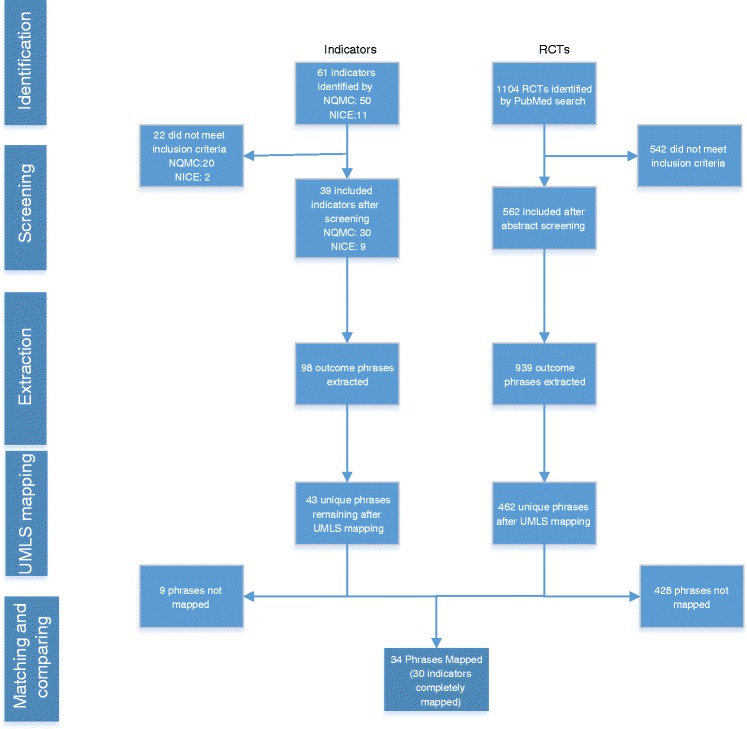
Flow diagram for the identification, extraction, and linking of indicators to outcome measures in randomised controlled trials

For this study we used a four-stage protocol for extracting indicators and linking them to outcome measures in the research literature (Fig. 1) [21]:

1. Identification and extraction of indicators from the USA and the UK;
2. Identification and extraction of outcome measures from RCTs;
3. Mapping indicators and RCT outcome measures to a standard clinical concept set within the Unified Medical Language System (UMLS), using the MetaMap tool [22];
4. Evaluating whether the mapped indicators could be associated with RCT outcome measures.

### Identification and extraction of indicators

National-level indicators for asthma management in children were collected from the US National Quality Measures Clearinghouse (NQMC) [23] and the UK’s NICE quality standard for asthma [24]. The NQMC was searched using the term “asthma” within the Respiratory Tract Diseases section on 5 March 2015. All documents identified by this process were then manually screened against inclusion criteria by two independent appraisers [MKC and DJ] and disagreements were resolved by consensus. Inter-rater agreement was measured using Cohen’s kappa [25].

The following inclusion criteria were used to select candidate indicators:

1. Any process or outcome indicators, AND
2. Any indicator of pharmacological or non-pharmacological management, AND
3. Applicable to children below 12 years of age, AND
4. Developed for national-level assessment.

Both appraisers identified all text phrases used to describe an indicator and then manually extracted these “indicator phrases”, resolving disagreements by consensus, using the following rules to normalise descriptions (Table 1, Additional file 1: Table S1):

**Table 1:** Examples of extracted indicator phrases

Indicator type	Extracted indicator phrase
*Recommended care*	“hospitalization”, “asthma action plan”
Medication	“steroid”, “inhaled corticosteroids”, “leukotriene inhibitors”
Event	“exacerbation of asthma”

1. Delete: Unit of analysis (e.g. number of people/patients)
2. Delete: Time points/frames (e.g. more than 24 h, within 2 days) for a process to occur
3. Delete: The term “asthma” if it describes patients
4. Extract: Any remaining noun phrases describing the unit of analysis.

### Identification and extraction of outcomes from RCTs

Any English language RCT on asthma management for children aged below 12 years of age and published in the last 10 years (2005–2014) was included in the study. The following search terms were used to retrieve PubMed articles (searched 3 December 2014):

1. Asthma
2. (randomised controlled trial[Publication Type] OR (randomised[Title/Abstract] AND controlled[Title/Abstract] AND trial[Title/Abstract]))
3. #1 AND #2
4. #3 AND “English”[Language]

PubMed filters for Species (“Humans”) and Ages (“Infant: birth-23 months”, “Preschool Child: 2–5 years”, and “Child: 6–12 years”) were also applied.

Two reviewers [MKC and DJ] independently screened the titles and abstracts of retrieved documents, using the following inclusion criteria:

1. Article describes an RCT (but excluding secondary/post-hoc analysis/protocol of RCTs);
2. The participants of the trial include children with asthma aged below 12 years of age, even if the trial also includes other participants;
3. The trial’s focus is on medical or non-medical management of asthma.

If an abstract did not contain enough information to make a decision, the full text was retrieved and assessed. Inter-rater agreement was measured and any disagreements were then resolved by consensus.

One appraiser [MKC] manually extracted all text phrases used to describe an outcome (“outcome phrases”) from included full text reports. For validation, a second appraiser [DJ] extracted outcome phrases from 50 randomly selected RCTs.

Outcome phrases were normalised using the following rules:

1. **Deduplication**: Similar outcome phrases mentioned repeatedly in an RCT were counted as a single occurrence.
2. **Specialisation**: Within a given RCT, the most specific version of an outcome phrase was preferred. For example, both *spirometry* and *peak flow measurement* describe lung function tests, but the latter is more specific.

### UMLS concept mapping using MetaMap

The list of extracted phrases for indicators and RCT outcome measures were then mapped to each other using a common set of standardised terms found in the Unified Medical Language System (UMLS) Metathesaurus, publicly available from the US National Library of Medicine. The Metathesaurus contains over 1 million biomedical concepts aggregated from over 100 source vocabularies.

The mapping was undertaken using a software tool created for the Metathesaurus called MetaMap [22]. MetaMap is well tested and has been used for many tasks, including information retrieval [26] and text mining [27, 28], and has been shown to perform well in mapping biomedical concepts to text. [29] The extracted indicator and outcome phrases were put into to MetaMap and all the UMLS concept outputs were recorded (Table 2). For example, the phrases ‘exacerbation of asthma’ and ‘asthma exacerbation’ both map to the UMLS concept C0349790 (Exacerbation of asthma). After the list of indicators and outcomes were re-expressed as UMLS concepts, a manual comparative analysis was undertaken looking for linkages between the two lists.

**Table 2:** Examples of UMLS concepts identified by MetaMap for given text phrases

Phrase	UMLS concepts
“exacerbation of asthma”	C0349790: Exacerbation of asthma [Finding]
	C0004096: Asthma (Asthma) [Disease or Syndrome]
	C2984299: Asthma (Asthma Pathway) [Functional Concept]
“Asthma exacerbation”	C0349790: Asthma exacerbation (Exacerbation of asthma) [Finding]
	C0004096: Asthma (Asthma) [Disease or Syndrome]
	C2984299: Asthma (Asthma Pathway) [Functional Concept]
“PEF”	C0030771: PEF (Pefloxacin) [Antibiotic,Organic Chemical]
	C1518922: PEF (Peak Expiratory Flow) [Laboratory Procedure]
	C1542834: PEF (Peak expiratory flow rate) [Finding]
“Peak expiratory flow”	C1518922: Peak Expiratory Flow [Laboratory Procedure]
	C0857465: Peak flow [Finding]
	C0231800: Expiratory (Expiration, function) [Organ or Tissue Function]
	C0444505: Peak (Peak level) [Quantitative Concept]
	C0806140: Flow (flow) [Natural Phenomenon or Process]

## Results

A total of 50 indicators were identified from the NQMC and 11 from NICE. Of these, 39 indicators were included (see Additional file 1: Table S2), with an inter-rater agreement of 0.895 (Cohen’s Kappa). Some 22 indicators were excluded because they were not associated with asthma management (15), were not process or outcome indicators (4), were not developed for national-level assessment (2) or did not apply to children below 12 years of age (2).

From year 2005–2014, 1104 RCTs were retrieved and after screening, 562 were included (Cohen’s Kappa = 0.7625). Validation of outcome measure extraction using a set of 50 randomly selected RCTs saw strong agreement (Cohen’s Kappa = 0.805).

The 39 indicators were described using 43 unique indicator phrases, and these were associated with 251 UMLS concepts. A total of 462 unique outcome phrases were identified from the RCT reports and these were associated with 1611 UMLS concepts.

**Fig. 2:**
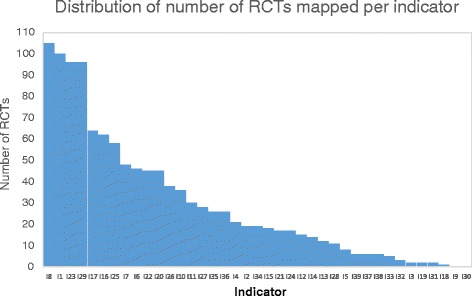
Number of RCTs that could be linked to each indicator, in descending order of frequency. (Additional file 1: Table S4 assigns each of the Indicator identification numbers on the X-axis with an indicator name)

For 30 of the 39 indicators (77%) all of their descriptive phrases could be fully mapped to outcome phrases. For 7 indicators (19%), at least some of their indicator phrases could be mapped to outcomes using MetaMap. Only two indicators (5%) could not be mapped to any RCT outcome (Fig. 2). The 30 fully mapped indicators were linked to outcomes via 34 phrases (Additional file 1: Table S3). Thus, about 95% of indicators (37/39) could be linked to at least some clinical trial reports using outcome measures, with a mean of 29.7 (Fig. 2, Additional file 1: Table S4). As Metamap was always able to generate concepts for indicators and outcomes, mapping failures were due to mismatches between the concepts assigned to indicators and to outcomes.

Not all of the phrases associated with these indicators were linked to outcome measures however, with only 34/43 (79%) mapping. Also, most outcome measures were never used as indicators, with only 34/462 (7%) of all outcome phrases linking to indicators.

## Discussion

The process of assessing the evidence base behind an indicator requires methods to identify the clinical trials that are likely to contain that evidence. Using a standardised protocol that matched terms and phrases for indicators and outcomes to concepts in the UMLS, we were able to successfully link the vast majority of indicators used to track the management of asthma in children to randomised controlled trials.

Our approach relies on the recognition that indicators are measures and that in clinical trial reports, outcome measures serve the same purpose. The next stage in assessing indicators would require an evaluation of the performance of the outcome measures used in the trial reports, and the nature of the indicator relationship with outcomes e.g. positively or negatively correlated. Outcome measures that are commonly used, that are relatively inexpensive and do not require specialised methods, and that repeatedly are able to distinguish the performance of different management approaches [30], are those that are likely to be favoured.

While there is a concern that many indicators are not based on RCT evidence, the indicators used in this study came from NQMC and NICE and were typically linked with references to the research literature. As such, they provided a gold standard to assess the effectiveness of the methods described here, which mapped terms used to describe indicators and outcomes to UMLS concepts, and then matched the indicators and outcomes with each other by finding common UMLS concepts. Our study shows that at least in the case of asthma management in children, the method is able in most cases to independently link indicator terms to clinical trial outcome terms, supporting its use when there is uncertainty about the evidence behind an indicator.

A strength of the protocol used here to link indicators to trials is that it lends itself to full automation. While the search for documents and extraction of text phrases was undertaken manually, both tasks readily lend themselves to automation using modern search and text processing computational methods. There is currently strong interest in automating many of the processes associated with systematic review [31, 32], and it is just as plausible to apply this approach to the process of indicator assessment.

### Strengths and limitations

To our knowledge, this is the first study to utilise a method that matches indicators with research evidence in clinical trials. Its strength is the use of a simple and systematic approach to identify candidate indicators using publicly available search and concept mapping tools, and the typically strong inter-rater agreements at each stage of the process. The use of the UMLS Metathesaurus and MetaMap allows indicators and outcome measures to be mapped in a repeatable and standard way.

We however only examined the link between indicators and RCT evidence, and other forms of evidence are likely to be used in the assessment of candidate indicators. Our method relies on the easy identification of outcome measures in trial reports, as these often use the PICO (Population, Intervention, Comparator, Outcome) structure in their abstract. Identification of outcome measures in other forms of evidence may thus need to depend on detailed screening of text in abstracts or full papers, which is likely to have higher error rates.

## Conclusions

Most of the terms used in quality indicators for asthma management in children can be linked to clinical trials using a robust method involving public resources, and this is the first stage in assessing the quality of indicators. The process of indicator development is a strong candidate for the use of automation tools, which have the potential to increase the quality and speed of indicator development.

## Additional file

Additional file 1: Additional Data Table S1. Examples of raw clinical indicators phrases, the normalised extracted form extracted, and their classification; **Table S2**: Number of included and excluded indicators (with reason) from NQMC and NICE; **Table S3**: Mapping phrases from indicators and RCTs; **Table S4**: The 39 included indicators, ordered with the number of RCTs mapped. (DOCX 27 kb)

## Abbreviations

BTS/SIGN: British Thoracic Society/Scottish Intercollegiate Guidelines Network
NICE: National Institute for Health and Care Excellence
NQMC: The US National Quality Measures Clearinghouse
RCT: Randomised controlled clinical trial
UMLS: Unified Medical Language System

## References

[1] Crampton P, Perera R, Crengle S (2004). What makes a good performance indicator? Devising primary care performance indicators for New Zealand. N Z Med J.

[2] Mainz J (2003). Defining and classifying clinical indicators for quality improvement. Int J Qual Health Care.

[3] Pencheon D (2008). The good indicators guide: understanding how to use and choose indicators: NHS Institute for Innovation and Improvement.

[4] Institute of Medicine (2006). Performance Measurement: Accelerating Improvement (Pathways to Quality Health Care Series).

[5] Wollersheim H, Hermens R, Hulscher M (2007). Clinical indicators: development and applications. Neth J Med.

[6] Hibbert P, Hannaford N, Long J, et al. Final Report: Performance indicators used internationally to report publicly on healthcare organisations and local health systems: Australian Institute of Health Innovation. Sydney: University of New South Wales; 2013.

[7] Campbell S, Braspenning J, Hutchinson A (2003). Improving the quality of health care: research methods used in developing and applying quality indicators in primary care. Br Med J.

[8] Kötter T, Blozik E, Scherer M (2012). Methods for the guideline-based development of quality indicators–a systematic review. Implement Sci.

[9] Woolf SH, Battista RN, Anderson GM (1990). Assessing the clinical effectiveness of preventive maneuvers: Analytic principles and systematic methods in reviewing evidence and developing clinical practice recommendations A report by the Canadian task force on the periodic health examination. J Clin Epidemiol.

[10] Sackett DL, Straus SE, Richardson WS, et al. Evidence-based medicine: how to practice and teach EBM. 2nd ed. Edinburgh: Churchill Livingstone; 2000.

[11] Cruz AA, Bousquet J, Khaltaev N. Global surveillance, prevention and control of chronic respiratory diseases: a comprehensive approach. Switzerland: World Health Organization; 2007.

[12] British Thoracic Society Scottish Intercollegiate Guidelines Network (2008). British guideline on the management of asthma. Thorax.

[13] National Asthma Council Australia (2014). Australian Asthma Handbook, Version 1.0.

[14] National Asthma Education Prevention Program (2007). Third Expert Panel on the Management of Asthma. Expert panel report 3: guidelines for the diagnosis and management of asthma: US Department of Health and Human Services, National Institutes of Health, National Heart, Lung, and Blood Institute.

[15] Australian Centre for Asthma Monitoring (2007). Australian asthma indicators: Five-year review of asthma monitoring in Australia.

[16] Praena-Crespo M, Ruiz-Canela J, Aquino N, Sanchez-Diaz J, García-Gestoso M. Evidence-based asthma indicators for primary care using RAND method. Eur Respir J. 2013;42(Suppl 57):P3834.

[17] Tanne J (2006). AMA develops measures of doctors’ performance. Br Med J.

[18] To T, Guttmann A, Lougheed MD (2010). Evidence-based performance indicators of primary care for asthma: a modified RAND Appropriateness Method. Int J Qual Health Care.

[19] Assurance NCfQ (2014). HEDIS 2014 Volume 2: Technical Update.

[20] Desai M, Oppenheimer JJ (2011). Medication adherence in the asthmatic child and adolescent. Curr Allergy Asthma Rep.

[21] Choong MK, Tsafnat G, Hibbert P, et al. Comparing clinical quality indicators for asthma management in children with outcome measures used in randomised controlled trials: a protocol. BMJ Open. 2015;5(9) doi: 10.1136/bmjopen-2015-008819. 10.1136/bmjopen-2015-008819 PMC4563246 26351189

[22] Aronson AR. Effective mapping of biomedical text to the UMLS Metathesaurus: the MetaMap program. Proc AMIA Symp. 2001;17–21. PMC2243666 11825149

[23] National Quality Measures Clearinghouse (NQMC). Agency for Healthcare Research and Quality (AHRQ) Rockville (MD) [Available from: http://www.qualitymeasures.ahrq.gov. 10.1080/15360280802537332 21923316

[24] NICE quality standards. National Institute for Health and Care Excellence (NICE) [Available from: https://www.nice.org.uk/standards-and-indicators.

[25] Cohen J (1960). A coefficient of agreement for nominal scales. Educ Psychol Meas.

[26] Choi S, Choi J, Yoo S (2014). Semantic concept-enriched dependence model for medical information retrieval. J Biomed Inform.

[27] Osborne JD, Lin S, Zhu LJ, et al. Mining biomedical data using MetaMap Transfer (MMtx) and the Unified Medical Language System (UMLS). Methods Mol Biol. 2007;408:153–69. 10.1007/978-1-59745-547-3_9 18314582

[28] Jimeno Yepes A, Berlanga R (2015). Knowledge based word-concept model estimation and refinement for biomedical text mining. J Biomed Inform.

[29] Pratt W, Yetisgen-Yildiz M (2003). A study of biomedical concept identification: MetaMap vs. People. AMIA Annu Symp Proc.

[30] Rothwell P (2000). Responsiveness of outcome measures in randomised controlled trials in neurology. J Neurol Neurosurg Psychiatry.

[31] Tsafnat G, Dunn A, Glasziou P, et al. The automation of systematic reviews. BMJ. 2013;346:f139. 10.1136/bmj.f139 23305843

[32] Tsafnat G, Glasziou P, Choong MK (2014). Systematic review automation technologies. Syst Rev.

