# Supplementary material for: Linking clinical quality indicators to research evidence - a case study in asthma management for children
Source: BMC Health Serv Res. 2017 Jul 21;17:502. doi: 10.1186/s12913-017-2324-y (PMC5521100; doi:10.1186/s12913-017-2324-y)
Supplement: Additional file 1: Additional Data Table S1. — Examples of raw clinical indicators phrases, the normalised extracted form extracted, and their classification; Table S2: Number of included and excluded indicators (with reason) from NQMC and NICE; Table S3: Mapping phrases from indicators and RCTs; Table S4: The 39 included indicators, ordered with the number of RCTs mapped. (DOCX 27 kb) [file 12913_2017_2324_MOESM1_ESM.docx]

### Additional file 1 – Additional Data

Table S1: Examples of raw clinical indicators phrases, the normalised extracted form extracted, and their classification.

| **A2** | **Extracted indicator phrase** | **Classification** |
| --- | --- | --- |
| Pediatric asthma inpatients who received **relievers** during **hospitalization**. | relievers;  hospitalization | Medication;  Type of recommended care |
| Pediatric **inpatient** **discharge**, age 2 years through 17 years, with an *International Classification of Diseases, Ninth Revision, Clinical Modification (ICD-9-CM) Principal Diagnosis Code* of asthma. | inpatient discharges | Type of recommended care |
|  |  |  |
| Number of asthma patients who have an **asthma** **discharge** **plan**. | asthma discharge plan | Type of recommended care |
| Number of asthma patients who were seen in an **emergency** **department** or **hospitalized** for asthma treatment. | emergency department; hospitalized | Type of recommended care;  Type of recommended care |
| The number of people in the denominator followed up by their own **GP** **practice** within 2 working days of treatment. | GP practice | Type of recommended care |
| The number of people who received treatment in **hospital** or through **out-of-hours** **services** for an **acute** **exacerbation** **of** **asthma**. | hospital;  out-of-hours services;  acute exacerbation of asthma | Type of recommended care;  Type of recommended care;  Event |

Table S2: Number of included and excluded indicators (with reason) from NQMC and NICE.

|  | **Reason** | **NQMC** | **NICE** |
| --- | --- | --- | --- |
| Included |  | 30 | 9 |
| Excluded | Not process or outcome indicators | 2 | 2 |
|  | Not asthma management | 15 | 0 |
|  | Not children aged below 12 years of age | 1 | 0 |
|  | Not developed for national-level assessment | 2 |  |
| Total |  | 50 | 11 |

Table S3: Mapping phrases from indicators and RCTs.

| Phrases from indicators | No of indicators | Phrases from RCTs | No of RCTs |
| --- | --- | --- | --- |
| Controller medication(s)  Control medication  Controller therapy | 6 | Controller medication(s)  Controller usage  Controller-medication use  Asthma controller medication | 6 |
| Discharged | 6 | Hospital discharge  Time to discharge  Time to hospital discharge  Time from ED triage to disposition decision  Disposition for the enrolled patients | 6 |
| Hospitalization  Hospitalized  Hospital  Readmitted to the hospital | 5 | Hospital days  Hospitalization(s)  Hospital admission  Hospital admission(s)  Admission to the hospital  Hospitalized | 56 |
| Exacerbation of asthma | 4 | Exacerbation(s)  Asthma exacerbation(s)  Exacerbations of asthma  Sever exacerbations  Exacerbation episodes | 40 |
| Medication  Asthma medications | 4 | Medication(s)  Asthma medications  Asthma medication use  Asthma drug use | 26 |
| Inhaled corticosteroids  Inhaled corticosteroid  Inhaler treatment | 3 | Inhaled corticosteroid(s)  UCS  UCS use  UCS dose | 17 |
| Asthma control | 3 | Asthma control | 16 |
| Asthma review  Structured review | 3 | Asthma review  Asthma evaluation | 2 |
| Peak flow measurement  Peak flow | 2 | Peak expiratory flow rates  PEFR  PEF  Peak expiratory flow | 96 |
| Emergency department | 2 | Emergency department visit  Visits to Accident and Emergency Department  Emergency room [ER] visits  ED visits  Emergency hospital visit(s)  Emergency room  Emergency visits  ED | 44 |
| Relievers | 2 | Reliever medication  Reliever medication use  Use of reliever medication  Relief medication | 9 |
| Oral or intravenous steroids  Systemic corticosteroids | 2 | Systemic corticosteroid(s)  Systemic corticosteroid use  Use of systemic corticosteroid | 6 |
| Respiratory symptoms  Exacerbation of respiratory symptoms | 2 | Respiratory symptoms  Respiratory signs  Respiratory tract symptoms | 5 |
| Written personalised action plan  Written Home Management Plan Care | 2 | Written asthma action plan  Written asthma management plan  Asthma action plans | 4 |
| Severity assessment  Measurement of severity | 2 | Asthma severity score | 3 |
| Inhaled anti-inflammatory medication  Anti-inflammatory medication | 2 | Inhaled steroids  Inhaled steroid-sparing effect | 2 |
| Short acting beta2 agonist inhalers  Short-acting beta2 agonist inhaler | 2 | Short-acting b2 adrenoceptor agnonists  Short-acting b-agonist use  SABA use | 4 |
| Seen by a clinician | 2 | Physician visit | 1 |
| Asthma treatment  Treatment | 2 | Treatments | 1 |
| Symptoms | 1 | Symptom(s)  Asthma symptoms  Signs/symptoms | 58 |
| Lost work or school days | 1 | Missed work and school days  School days missed  Days off school  School absences  School absenteeism  Absenteeism  Absent from school  School/work absence  Time off school/nursery  Absence from work  School and work absences | 38 |
| Symptom-free days | 1 | Symptom-free 24h periods  Symptom-free day(s)  Symptom-free days (SFDs) | 28 |
| Allergy testing | 1 | Allergen-induced skin responses  Titrated SPT  Skin prick tests | 10 |
| GP practice | 1 | Visits to general practitioner  General practitioner (GP) visits  PCP visit  Visits to the PCP  Primary care contacts | 9 |
| Self-management goal | 1 | Asthma self-management  Asthma self-assessment score | 5 |
| Control of other triggers | 1 | Trigger exposure and control  Efforts to control asthma triggers  Action taken to reduce exposure to asthma triggers | 4 |
| Follow-up care | 1 | Follow-up visit  Follow-up with a primary care provider | 2 |
| Rescue action | 1 | Need for rescue therapy  Need for rescue | 2 |
| Screening for depression | 1 | Hospital Anxiety and Depression Scale anxiety subscale | 2 |
| Training and assessment in inhaler technique | 1 | Inhaler technique | 2 |
| Change in medication or clinical status | 1 | Changes in medications | 1 |
| Environmental control | 1 | Compliance on environmental control measures | 1 |
| Environment triggers | 1 | Home environmental markers | 1 |
| Questionnaire | 1 | questionnaire | 1 |

Table S4: The 39 included indicators, ordered with the number of RCTs mapped.

|  | Quality measure | Numerator | Denominator | No. RCT mapped |
| --- | --- | --- | --- | --- |
| I8 | People who received treatment in hospital or through out-of-hours services for an acute exacerbation of asthma are followed up by their own GP practice within 2 working days of treatment. | the number of people in the denominator followed up by their own GP practice within 2 working days of treatment | the number of people who received treatment in hospital or through out-of-hours services for an acute exacerbation of asthma | 105 |
| I1 | People with asthma receive a written personalised action plan. | a) the number of people in the denominator receiving a written personalised action plan b) the number of people in the denominator receiving a written personalised action plan before discharge | a) the number of people with asthma b)the number of people treated in hospital for an acute exacerbation of asthma | 100 |
| I23 | Diagnosis and management of asthma: percentage of patients with spirometry or peak flow at the last visit related to asthma. | Number of asthma patients who had spirometry or peak flow measurement at the last visit related to asthma | Number of asthma patients seen for an asthma related visit (see the related "Denominator Inclusions/Exclusions" field) | 96 |
| I29 | Asthma: percent of patients older than 5 years with moderate or severe persistent asthma who have established a "personal best" peak flow. | The number of patients from the denominator who have established a "personal best" peak flow through multiple measurements during a period of relative disease stability (see the related "Numerator Inclusions/Exclusions" field) | Total number of patients with a National Heart, Lung, and Blood Institute (NHLBI) classification of moderate or severe persistent asthma older than five years in the registry | 96 |
| I17 | Diagnosis and management of asthma: percentage of hospitalized patients with asthma who are discharged on an inhaled anti-inflammatory medication. | Number of asthma patients who are discharged on an inhaled anti-inflammatory medication | Number of asthma patients who were hospitalized (see the related "Denominator Inclusions/Exclusions" field) | 64 |
| I16 | Diagnosis and management of asthma: percentage of discharged patients with asthma who are readmitted to hospital within 30 days of discharge. | Number of asthma patients who are readmitted to the hospital within 30 days of discharge from an asthma-related hospitalization | Number of asthma patients who were discharged from an asthma-related hospitalization (see the related "Denominator Inclusions/Exclusions" field) | 62 |
| I25 | Asthma: percentage of patients aged 5 to 40 years with diagnosed asthma who were evaluated during at least one office visit during the reporting year for the frequency (numeric) of daytime and nocturnal asthma symptoms. | Patients in the denominator who were evaluated during at least one office visit during the reporting year for the frequency (numeric) of daytime and nocturnal asthma symptoms | All patients aged 5 to 40 years with diagnosed asthma | 58 |
| I7 | People admitted to hospital with an acute exacerbation of asthma have a structured review by a member of a specialist respiratory team before discharge. | the number of people in the denominator receiving a structured review by a member of a specialist respiratory team | the number of people discharged from hospital after admission for an acute exacerbation of asthma | 48 |
| I6 | People aged 5 years or older presenting to a healthcare professional with a severe or life-threatening acute exacerbation of asthma receive oral or intravenous steroids within 1 hour of presentation. | the number of people in the denominator receiving oral or intravenous steroids within 1 hour of presentation | the number of people aged 5 years or older presenting to a healthcare professional with a severe or life-threatening acute exacerbation of asthma | 46 |
| I22 | Diagnosis and management of asthma: percentage of patients with asthma who return to the emergency department for treatment of asthma within 30 days of last visit to the emergency department. | Number of asthma patients who return to the emergency department for treatment of asthma within 30 days of the last visit to the emergency department | Number of asthma patients who were seen in the emergency department for asthma treatment (see the related "Denominator Inclusions/Exclusions" field) | 45 |
| I20 | Diagnosis and management of asthma: percentage of patients with an emergency department visit or inpatient admission for an asthma exacerbation who are discharged from the emergency department or inpatient setting with an asthma discharge plan. | Number of asthma patients who have asthma discharge plan | Number of asthma patients who were seen in emergency department or hospitalized for asthma treatment (see the related "Denominator Inclusions/Exclusions" field) | 45 |
| I26 | Asthma: average number of lost workdays and/or school days in the past 30 days. | The sum of the number of days in the past 30 lost at work or school because of asthma over all patients who report lost work or school days | Total number of patients with asthma in the registry who have been queried about lost work or school days at last contact | 38 |
| I10 | Medication therapy for persons with asthma: percentage of patients with asthma during the measurement period who were dispensed more than 3 canisters of short acting beta2 agonist inhalers over a 90-day period and who did not receive controller therapy during the same 90-day period (absence of controller therapy). | The number of patients with persistent asthma during the measurement period who were dispensed more than 3 canisters of short acting beta2 agonist inhalers over a 90-day period and who did not receive controller therapy during the same 90-day period (see the related "Numerator Inclusions/Exclusions" field) | Patients 5 to 50 years with consecutive fills of asthma medications during the measurement year (see the related "Denominator Inclusions/Exclusions" field) | 36 |
| I11 | Medication therapy for persons with asthma: percentage of patients with persistent asthma who were dispensed more than 3 canisters of a short-acting beta2 agonist inhaler during the same 90-day period (suboptimal control). | The number of patients with persistent asthma who were dispensed more than 3 canisters of a short-acting beta2 agonist inhaler during the same 90-day period (see the related "Numerator Inclusions/Exclusions" field) | Patients 5 to 50 years with consecutive fills of asthma medications during the measurement year (see the related "Denominator Inclusions/Exclusions" field) | 30 |
| I27 | Asthma: average number of symptom-free days in the previous two weeks. | The sum of the number of symptom-free days in the previous two weeks over all patients in the denominator (see the related "Numerator Inclusions/Exclusions" field) | Total number of patients with asthma in the registry who report symptom-free days | 28 |
| I35 | Use of appropriate medications for people with asthma: percentage of patients 5 to 64 years of age during the measurement year who were identified as having persistent asthma and who were appropriately dispensed medication during the measurement year. | Evidence of at least one asthma medication for a preferred therapy during the measurement year (see the related "Numerator Inclusions/Exclusions" field) | Patients age 5 to 64 years by December 31 of the measurement year who were identified as having persistent asthma (see the related "Denominator Inclusions/Exclusions" field) | 26 |
| I36 | Asthma medication ratio: percentage of members 5 to 64 years of age who were identified as having persistent asthma and had a ratio of controller medications to total asthma medications of 0.50 or greater during the measurement year. | The number of members who have a medication ratio of 0.50 or greater during the measurement year (see the related "Numerator Inclusions/Exclusions field) | Members 5 to 64 years of age by December 31 of the measurement year with persistent asthma (see the related "Denominator Inclusions/Exclusions" field) | 26 |
| I4 | People with asthma who present with respiratory symptoms receive an assessment of their asthma control. | the number of people in the denominator receiving an assessment of their asthma control | the number of people with asthma who present with respiratory symptoms | 21 |
| I2 | People with asthma are given specific training and assessment in inhaler technique before starting any new inhaler treatment. | the number of people in the denominator who have training and assessment in inhaler technique | the number of people with asthma starting a new inhaler treatment | 19 |
| I34 | Asthma: percent of patients with persistent asthma at last contact who are on an anti-inflammatory medication. | The number of patients from the denominator who are on anti-inflammatory medication (Medications include chronically administered inhaled corticosteroids, mast cell stabilizers and leukotriene inhibitors.) | Total number of patients with an underlying National Heart, Lung, and Blood Institute (NHLBI) classification of persistent asthma at last contact in the registry | 19 |
| I15 | Asthma: the percentage of patients with asthma, on the register, who have had an asthma review in the preceding 12 months that includes an assessment of asthma control using the 3 RCP questions. | Number of patients from the denominator who have had an asthma review in the preceding 12 months that includes an assessment of asthma control using the 3 Royal College of Physicians (RCP) questions (see the related "Numerator Inclusions/Exclusions" field) | Patients with asthma, on the register (see the related "Denominator Inclusions/Exclusions" field) | 18 |
| I21 | Diagnosis and management of asthma: percentage of patients with assessment of asthma control using a validated questionnaire at the last visit related to asthma. | Number of asthma patients who had an assessment of asthma control using a validated questionnaire | Number of asthma patients seen for an asthma related visit (see the related "Denominator Inclusions/Exclusions" field) | 17 |
| I24 | Asthma: percentage of patients aged 5 to 40 years diagnosed with mild, moderate, or severe persistent asthma who were prescribed either the preferred long-term control medication (inhaled corticosteroid) or an acceptable alternative treatment. | Patients in the denominator who were prescribed either the preferred long-term control medication (inhaled corticosteroid) or an acceptable alternative treatment | All patients aged 5 to 40 years with diagnosed mild, moderate, or severe persistent asthma | 17 |
| I12 | Children's asthma care: percent of pediatric asthma inpatients who received relievers during hospitalization. | Pediatric asthma inpatients who received relievers during hospitalization | Pediatric asthma inpatients (age 2 years through 17 years) who were discharged with a principal diagnosis of asthma (see the related "Denominator Inclusions/Exclusions" field) | 15 |
| I14 | Children's asthma care: percent of pediatric asthma inpatients with documentation that they or their caregivers were given a written Home Management Plan of Care (HMPC) document. | Pediatric asthma inpatients with documentation that they or their caregivers were given a written Home Management Plan of Care (HMPC) document that addresses all of the following: 1) Arrangements for follow-up care 2) Environmental control and control of other triggers 3) Method and timing of rescue actions 4) Use of controllers 5) Use of relievers | Pediatric asthma inpatients (age 2 years through 17 years) discharged home (see the related "Denominator Inclusions/Exclusions" field) | 14 |
| I13 | Children's asthma care: percent of pediatric asthma inpatients who received systemic corticosteroids during hospitalization. | Pediatric asthma inpatients who received systemic corticosteroids during hospitalization | Pediatric asthma inpatients (age 2 years through 17 years) who were discharged with a principal diagnosis of asthma (see the related "Denominator Inclusions/Exclusions" field) | 12 |
| I28 | Asthma: percent of patients evaluated for environmental triggers other than environmental tobacco smoke (dust mites, cats, dogs, molds/fungi, cockroaches) either by history of exposure and/or by allergy testing. | The number of patients from the denominator evaluated for environmental triggers other than environmental tobacco smoke (dust mites, cats, dogs, molds/fungi, cockroaches) either by history of exposure and/or by allergy testing | Total number of patients with asthma in the registry | 11 |
| I5 | People with asthma who present with an exacerbation of their symptoms receive an objective measurement of severity at the time of presentation. | the number of people in the denominator receiving an objective measurement of severity at the time of presentation | the number of people with asthma presenting with an exacerbation of their respiratory symptoms | 8 |
| I39 | Use of appropriate medications for people with asthma: percentage of members 5 to 64 years of age during the measurement year who were identified as having persistent asthma and who were appropriately prescribed medication during the measurement year. | Dispensed at least one prescription for an asthma controller medication during the measurement year (see the related "Numerator Inclusions/Exclusions" field) | Members 5 to 64 years of age by December 31 of the measurement year with persistent asthma (see the related "Denominator Inclusions/Exclusions" field) | 6 |
| I37 | Medication management for people with asthma: percentage of members 5 to 64 years of age during the measurement year who were identified as having persistent asthma and who were dispensed an asthma controller medication that they remained on for at least 50% of their treatment period. | The number of members who achieved a proportion of days covered (PDC) of at least 50% for their asthma controller medications during the measurement year (see the related "Numerator Inclusions/Exclusions" field) | Members 5 to 64 years of age by December 31 of the measurement year with persistent asthma (see the related "Denominator Inclusions/Exclusions" field) | 6 |
| I38 | Medication management for people with asthma: percentage of members 5 to 64 years of age during the measurement year who were identified as having persistent asthma and who were dispensed an asthma controller medication that they remained on for at least 75% of their treatment period. | The number of members who achieved a proportion of days covered (PDC) of at least 75% for their asthma controller medications during the measurement year (see the related "Numerator Inclusions/Exclusions" field) | Members 5 to 64 years of age by December 31 of the measurement year with persistent asthma (see the related "Denominator Inclusions/Exclusions" field) | 6 |
| I33 | Asthma: percent of patients with documented self-management goals in the last 12 months. | The number of patients from the denominator with documented self-management goals in the last 12 months | Total number of patients with asthma in the registry | 5 |
| I32 | Asthma: percent of patients with a severity assessment at last contact (visit or phone). | The number of patients from the denominator with a severity assessment at last contact (visit or phone) | Total number of patients with asthma in the registry | 3 |
| I3 | People with asthma receive a structured review at least annually. | the number of people in the denominator who had a structured review within 12 months of the last review or diagnosis | the number of people with asthma | 2 |
| I19 | Diagnosis and management of asthma: percentage of patients whose asthma is not controlled or have change in medication or clinical status, who are seen by a health care clinician within two to six weeks. | Number of asthma patients who are seen by a clinician within two to six weeks of change in medication or clinical status | Number of asthma patients who are uncontrolled or have a change in medication or clinical status (see the related "Denominator Inclusions/Exclusions" field) | 2 |
| I31 | Asthma: percent of patients with a documented screening for depression in the past 12 months. | The number of patients from the denominator with a documented screening for depression in the past 12 months | Total number of patients with asthma in the registry | 2 |
| I18 | Diagnosis and management of asthma: percentage of patients whose asthma is controlled who are seen by a health care clinician every one to six months. | Number of asthma patients who are seen by a clinician every one to six months | Number of asthma patients who are controlled (see the related "Denominator Inclusions/Exclusions" field) | 1 |
| I9 | People with difficult asthma are offered an assessment by a multidisciplinary difficult asthma service. | the number of people in the denominator receiving an assessment by a multidisciplinary difficult asthma service | the number of people with difficult asthma | 0 |
| I30 | Asthma: percent of patients who have a record of influenza immunization in the past 12 months. | The number of patients from the denominator who have a record of influenza immunization in the past 12 months | Total number of patients with asthma in the registry | 0 |
